# Proton pump inhibitor treatment aggravates bacterial translocation in patients with advanced cirrhosis and portal hypertension

**DOI:** 10.1128/mbio.00492-23

**Published:** 2023-08-25

**Authors:** Lukas Sturm, Misa Hirose, Laura Stolz, Michael Schultheiss, Katharina Zoldan, Marlene Reincke, Jan Patrick Huber, Rafael Kaeser, Tobias Boettler, Robert Thimme, Elisabeth Albert, Hauke Busch, Axel Künstner, Dominik Bettinger

**Affiliations:** 1 Department of Medicine II, Faculty of Medicine, Medical Center University of Freiburg, Freiburg, Germany; 2 Berta-Ottenstein-Programme, Faculty of Medicine, University of Freiburg, Freiburg, Germany; 3 Luebeck Institute of Experimental Dermatology, University of Luebeck, Luebeck, Germany; 4 IMM-PACT-Programme, Faculty of Medicine, University of Freiburg, Freiburg, Germany; 5 Institute for Cardiogenetics, University of Luebeck, Luebeck, Germany; Rutgers University, Piscataway, New Jersey, USA

**Keywords:** bacterial translocation, cirrhosis, portal hypertension, proton pump inhibitor, microbiome

## Abstract

Recent studies have linked proton pump inhibitor (PPI) treatment to increased complications of cirrhosis, such as bacterial infections and hepatic encephalopathy. However, the underlying pathophysiological mechanisms remain unclear. The present study investigated the hypothesis that PPI treatment may promote adverse effects in patients with advanced cirrhosis by affecting subclinical bacterial translocation (BT) from the gut into the portal venous bloodstream. Blood samples from the portal vein were obtained during implantation of a transjugular intrahepatic portosystemic shunt (TIPS) in a total of 80 cirrhosis patients with PPI treatment (PPI group, *n* = 57) and without PPI treatment (no-PPI group, *n* = 23). BT was identified using a 16S ribosomal RNA gene (V1V2 region) polymerase chain reaction. The microbiota composition in the portal venous blood samples was further analyzed by deep amplicon sequencing. Indeed, the prevalence of BT was significantly higher in the PPI group compared to the no-PPI group (86.0% vs 52.2%, *P* = 0.001). Importantly, this effect was not attributable to group differences in the severity of cirrhosis, parameters of portal hypertension, or medication. Microbiome analyses showed significantly increased alpha-diversity (Shannon) in the portal venous blood of the PPI group. Taxonomic analyses revealed significantly increased *Streptococcus* abundances in these patients. The present study reveals aggravated BT in patients with advanced cirrhosis and portal hypertension receiving PPI therapy. Increased BT could be an important pathomechanism contributing to the adverse effects of PPI treatment in patients with cirrhosis.

**IMPORTANCE:**

Long-term prescription of proton pump inhibitors (PPIs) in patients with cirrhosis is common practice. However, in recent years, several observational studies have reported increased complications and negative prognostic effects of PPI treatment in these patients. Judging the significance of these associations is complicated by the fact that a plausible underlying pathomechanism has not been identified so far. In the present study, we address this important issue by investigating the impact of PPI treatment on subclinical bacterial translocation from the gut into the blood stream in patients with advanced cirrhosis and portal hypertension. Indeed, we report significantly aggravated bacterial translocation in cirrhosis patients receiving PPI treatment. This finding is highly relevant, as bacterial translocation is known to promote the development of complications and impair prognosis in patients with cirrhosis. Hence, the present study could establish a plausible link between PPI treatment and adverse effects in cirrhosis.

## INTRODUCTION

Treatment with proton pump inhibitors (PPIs) is common in patients with cirrhosis—of note, often as long-term medication without clear indication (1 – 3). In recent years, several observational studies have linked PPI treatment to increased complications in patients with advanced cirrhosis. This involves a higher incidence of bacterial infections, such as spontaneous bacterial peritonitis and pneumonia, as well as increased rates of hepatic encephalopathy (4 – 9). Further, some studies report (dose-dependent) impaired survival in PPI-treated patients (1, 10). It has to be mentioned, though, that some studies could not reproduce associations of PPI treatment with adverse events in cirrhosis (11, 12). Hence, negative effects of PPI treatment in patients with cirrhosis remain a controversial topic. This issue is complicated by the fact that, so far, the pathomechanisms underlying a possible connection between PPI treatment and adverse effects in cirrhosis have not been identified. However, previous studies have shown that PPI treatment can induce intestinal dysbiosis and promote small intestinal overgrowth (SIBO) in human beings in general and in patients with cirrhosis in particular (13 – 18). Of note, alterations in the gut microbiota composition, among other factors such as portal hypertension, are considered to play an important role in the development of bacterial translocation (BT) in advanced chronic liver disease (19). In consideration of these facts, we hypothesized that PPI treatment could have a significant impact on BT in patients with cirrhosis. Importantly, BT is a central pathomechanism of advanced cirrhosis that promotes the development of complications and is associated with impaired prognosis (20 – 22). Hence, increased BT could establish a plausible link between PPI treatment and adverse effects in cirrhosis. Against this background, the present study aimed to investigate the impact of PPI therapy on BT from the gut into the portal venous bloodstream in patients with cirrhosis.

## MATERIALS AND METHODS

### Patient selection and assessment of PPI treatment

**Fig 1:**
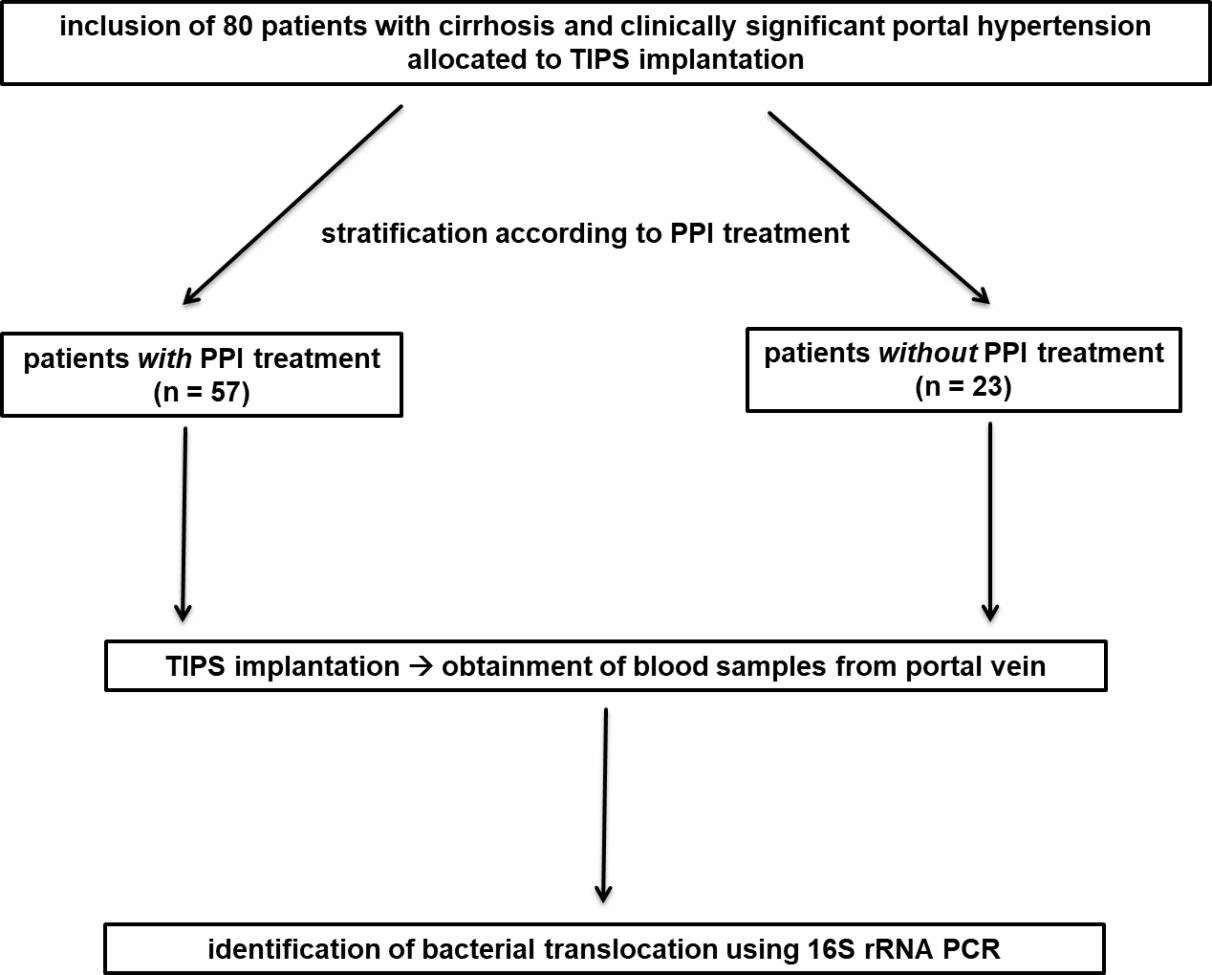
Eighty patients with cirrhosis and clinically significant portal hypertension allocated to TIPS implantation were included and stratified according to whether they received PPI treatment (PPI group: *n* =57, no-PPI group: *n* = 23). Subsequently, blood samples from the portal vein were obtained during TIPS placement and BT was identified by performing a 16S rRNA PCR in the blood samples.Abbreviations: BT, bacterial translocation; PCR, polymerase chain reaction; PPI, proton pump inhibitor; rRNA, ribosomal ribonucleic acid; TIPS, transjugular intrahepatic portosystemic shunt.

Patient selection and study design are summarized in Fig. 1. Eighty patients with liver cirrhosis and clinically significant portal hypertension were recruited during inpatient treatment for implantation of a transjugular intrahepatic portosystemic shunt (TIPS) at the Medical Center University of Freiburg, Germany, between January 2018 and October 2020. The patients’ demographic, clinical, laboratory, radiological, and interventional data were recorded. In all included patients, the diagnosis of liver cirrhosis was confirmed by pathognomonic findings on ultrasound examination and complementary liver stiffness measurement in case of inconclusive findings and/or by biopsy. TIPS implantation was indicated following the guidelines for the management of decompensated cirrhosis by the European Association for the Study of the Liver (EASL) (23). Severity of cirrhosis was assessed by the Model for End-Stage Liver Disease (MELD) and the Freiburg Index of Post-TIPS Survival (FIPS) that were calculated based on laboratory parameters recorded within 24 h prior to TIPS implantation. The presence of clinically significant portal hypertension [portosystemic pressure gradient (PSG) ≥ 10 mmHg] was confirmed during the TIPS intervention, prior to stent implantation, in all patients. All patients showed no clinical signs of infection at the time of TIPS implantation. On study inclusion, special focus was put on recording PPI medication. Patients were stratified into two groups: patients with PPI treatment, defined as daily PPI intake started at least 4 weeks prior to TIPS implantation (PPI group: *n* = 57, 71.3%) and patients who had not received PPI treatment for at least 4 weeks prior to TIPS implantation (no-PPI group: *n* = 23, 28.7%). In all patients with PPI treatment, PPI medication had been prescribed by physician discretion, independent of study participation. Indication, substance, and daily PPI dose at TIPS implantation were noted. Pantoprazole dose served as reference, meaning the daily dose of esomeprazole and omeprazole equaled double the pantoprazole dose.

### Blood sample collection

Blood samples from the portal vein were obtained during TIPS implantation in all patients. TIPS implantation was performed as described previously (24). In summary, a hepatic vein was catheterized via a transjugular approach. Subsequently, a transhepatic puncture of the right intrahepatic branch of the portal vein was performed under ultrasound guidance. A catheter was advanced into the main trunk of the portal vein, and blood samples were collected in EDTA tubes. Afterward, TIPS implantation was continued. TIPS implantation and handling of blood samples were performed under strictly sterile conditions. All patients received analgosedation with propofol and midazolam. Blood samples were centrifuged immediately after collection, and plasma aliquots were stored at −80°C until conduction of the analyses.

### Identification of BT

Bacterial DNA was isolated from the plasma samples using the MolYsis Complete5 kit by Molzym GmbH & Co KG, Bremen, Germany, which has been validated previously (25). The hyper-variable V1V2 region of the bacterial 16S ribosomal RNA (rRNA) gene was amplified by polymerase chain reaction (PCR) using the 27F/338R primer combination as described previously (26). The PCR product was analyzed using a 1.5% agarose gel electrophoresis, and BT was identified by the presence of PCR product. The PCR product was further processed to prepare for the sequencing library, and the final library was sequenced on the Illumina MiSeq platform with v2 chemistry (2 × 250 bp) as described previously (26). Obtained sequencing data were analyzed to define the microbiota composition in the portal venous blood. Further, levels of lipopolysaccharide binding protein (LBP), soluble cluster of differentiation 14 (sCD14), tumor necrosis factor-alpha (TNF-alpha), and interleukin-6 (IL-6) were analyzed, as these parameters have been proposed as surrogate markers of BT previously (27, 28). These parameters were determined using enzyme-linked immunosorbent assays by Cloud-Clone Corp., Texas, USA (SEB406Hu) and R&D Systems Inc., Minnesota, USA (DC140, DTA00D, D6050). Sample preparation and conduction of the assays were performed according to the manufacturers’ recommendations. Sequencing data used for this study were submitted to the European Nucleotide Archive (ENA) and are available under accession number PRJEB53045.

### Statistical analyses

Categorial variables were expressed as frequency and percentage, and continuous variables as median with interquartile range. The Kolmogorov-Smirnov test revealed no Gaussian distribution of the data. Group differences were determined by Chi square tests or Mann-Whitney U tests as appropriate. For microbiome analyses, alpha-diversity was assessed using the sample-wise and group-wise estimate of Shannon index, and beta-diversity was assessed using Aitchison distance (29). Taxonomic group differences on phylum or genus level were evaluated using a linear modeling approach and a likelihood ratio test. Detailed information on statistical methods and data processing for microbiome analyses is given in supplementary file 1. A *P*-value below 0.05 was considered significant. Statistical analyses were performed using SPSS (version 28.0, IBM, New York, USA), GraphPad Prism (version 9.3, GraphPad Software, California, USA), and R (version 4.2).

## RESULTS

### Patient characteristics

Patient characteristics at study inclusion are summarized in Table 1. PPI and no-PPI group were comparable with respect to age, gender distribution, and etiology of chronic liver disease, with alcoholic liver disease being the leading cause of cirrhosis. The two patient groups were also comparable with respect to laboratory parameters of liver function and clinical characteristics of cirrhosis, such as the presence of ascites and history of hepatic encephalopathy. However, a history of SBP was more frequent in the no-PPI group in comparison to the PPI group, albeit the difference was not statistically significant (*n* = 7, 30.4% vs *n* = 9, 15.8%; *P* = 0.138). PPI group and no-PPI group were in comparable stages of cirrhosis, as assessed by FIPS, MELD, and Child-Pugh stage. Refractory ascites was the leading indication for TIPS implantation in both patient groups. The degree of portal hypertension, as determined by PSG measurement prior to TIPS placement, was also comparable in both patient groups. Patients in the no-PPI group received medication with norfloxacin significantly more frequently (*n = 6*, 26.1% vs *n* = 4, 7.0%; *P* = 0.020). Otherwise, there was no difference between the patient groups with respect to medication other than PPIs possibly affecting BT, such as rifaximin or recent systemic antibiotic treatment. In the PPI group, a majority of patients received pantoprazole treatment with a daily dose of 40 mg. Of note, there was no clear indication for PPI treatment in 63.2% of patients.

**TABLE 1:** Patient characteristics of PPI group versus no-PPI group^*h*^

	Total of patients (*n* = 80)	No PPI (*n* = 23)	PPI (*n* = 57)	*P*-value
**Age [years]**	62 (56–70)	66 (58–72)	60 (56–69)	0.219
**Gender**				0.198
Female	22 (27.5)	4 (17.4)	18 (31.6)	
Male	58 (72.5)	19 (82.6)	39 (68.4)	
**Etiology**				0.758
Alcoholic	53 (66.3)	16 (69.6)	37 (64.9)	
HCV	8 (10.0)	2 (8.7)	6 (10.5)	
HBV	4 (5.0)	0	4 (7.0)	
NASH	9 (11.3)	3 (13.0)	6 (10.5)	
Other	6 (7.5)	2 (8.7)	4 (7.0)	
**Ongoing alcohol consumption**	17 (21.3)	5 (21.7)	12 (21.1)	0.994
**Ascites**	72 (90.0)	22 (95.7)	50 (87.7)	0.284
**Prior HE**	17 (21.3)	4 (17.4)	13 (22.8)	0.592
**Prior SBP**	16 (20.0)	7 (30.4)	9 (15.8)	0.138
**TIPS indication**				0.506
Ascites^*a*^	69 (86.3)	21 (91.3)	48 (84.2)	
Secondary prophylaxis^*b*^	8 (10.0)	2 (8.7)	6 (10.5)	
Other^*c*^	3 (3.8)	0	3 (5.3)	
**PSG pre-TIPS [mmHg]**	18 (16–21)	19 (17–23)	18 (16– 21)	0.160
**FIPS**	0.18 (−0.51–0.64)	0.22 (−0.52–1.00)	0.17 (−0.50–0.62)	0.602
**FIPS risk group**				0.202
High-risk (≥ 0.92)	17 (21.3)	7 (30.4)	10 (17.5)	
Low-risk (< 0.92)	63 (78.8)	16 (69.6)	47 (82.5)	
**MELD**	13 (10– 16)	15 (9– 17)	13 (10– 15)	0.620
**Child-Pugh stage**				0.828
A	11 (13.8)	4 (17.4)	7 (12.3)	
B	48 (60.0)	13 (56.5)	35 (61.4)	
C	21 (26.3)	6 (26.1)	15 (26.3)	
**Laboratory parameters**				
WBC [10^3^ /µL]	6 (5–8)	6 (4–9)	6 (5–8)	0.431
Platelets [10^3^ /µL]	125 (92–197)	109 (83–167)	133 (98–202)	0.184
Creatinine [mg/dL]	1.3 (0.9–1.9)	1.4 (0.9–2.2)	1.3 (0.9–1.9)	0.610
INR	1.2 (1.1–1.3)	1.2 (1.1–1.3)	1.2 (1.1–1.3)	0.932
Bilirubin [mg/dL]	1.1 (0.7–2.3)	1.1 (0.9–1.8)	1.1 (0.7–2.5)	0.798
Albumin [g/dL]	3.1 (2.8–3.4)	3.0 (2.8–3.3)	3.1 (2.7–3.5)	0.978
AST [U/L]	45 (30– 64)	47 (29– 58)	42 (30– 66)	0.974
ALT [U/L]	25 (17– 36)	32 (22– 48)	24 (16– 32)	0.023
Sodium [mmol/L]	135 (131– 138)	132 (129 –138)	136 (132 –138)	0.246
**Medication**				
NSBB	39 (36.3)	8 (34.8)	21 (36.8)	0.862
Norfloxacin	10 (12.5)	6 (26.1)	4 (7.0)	0.020
Rifaximin	13 (16.3)	5 (21.7)	8 (14.0)	0.398
Lactulose	44 (55.0)	12 (52.2)	32 (56.1)	0.747
Antibiotic treatment^*d*^	22 (27.5)	5 (21.7)	17 (29.8)	0.464
**PPI indication**				
Gastritis^*e*^			9 (15.8)	
Gastroduodenal ulcer^*e*^			3 (5.3)	
GERD^*f*^			9 (15.8)	
No clear indication			36 (63.2)	
PPI substance				
Pantoprazole			55 (96.5)	
Omeprazole			1 (1.8)	
Esomeprazole			1 (1.8)	
**PPI daily dose^*g*^**				
20 mg			8 (14.0)	
40 mg			39 (68.4)	
80 mg			10 (17.5)	

^*a*^ Refractory ascites including hepatic hydrothorax.

^*b*^ Secondary prophylaxis of variceal bleeding after failure of endoscopic and medicamentous treatment.

^*c*^ Preemptive TIPS within 72 h after variceal hemorrhage in two patients and portal decompression prior to planned abdominal surgery in one patient.

^*d*^ Systemic antibiotic treatment within 4 weeks prior to TIPS implantation.

^*e*^ Gastritis or gastroduodenal ulcer on endoscopy within 8 weeks prior to study inclusion.

^*f*^ Reflux esophagitis on endoscopy within 8 weeks prior to TIPS implantation and/or Barrett’s esophagus.

^*g*^ Pantoprazole dose served as reference, meaning daily doses of omeprazole and esomeprazole equaled double the pantoprazole dose.

^*h*^ ALT – alanine aminotransferase, AST aspartate aminotransferase, HBV/HCV – hepatitis B/C virus, FIPS – Freiburg Index of Post-TIPS Survival, GERD – gastroesophageal reflux disease, HE – hepatic encephalopathy, INR – international normalized ratio, MELD – Model for End-Stage Liver Disease, NASH – non-alcoholic steatohepatitis, NSBB – non-selective beta blocker, PPI – proton pump inhibitor, PSG – portosystemic pressure gradient, SBP – spontaneous bacterial peritonitits, TIPS – transjugular intrahepatic portosystemic shunt, WBC – white blood cells.

### Aggravated BT in the PPI group

**Fig 2:**
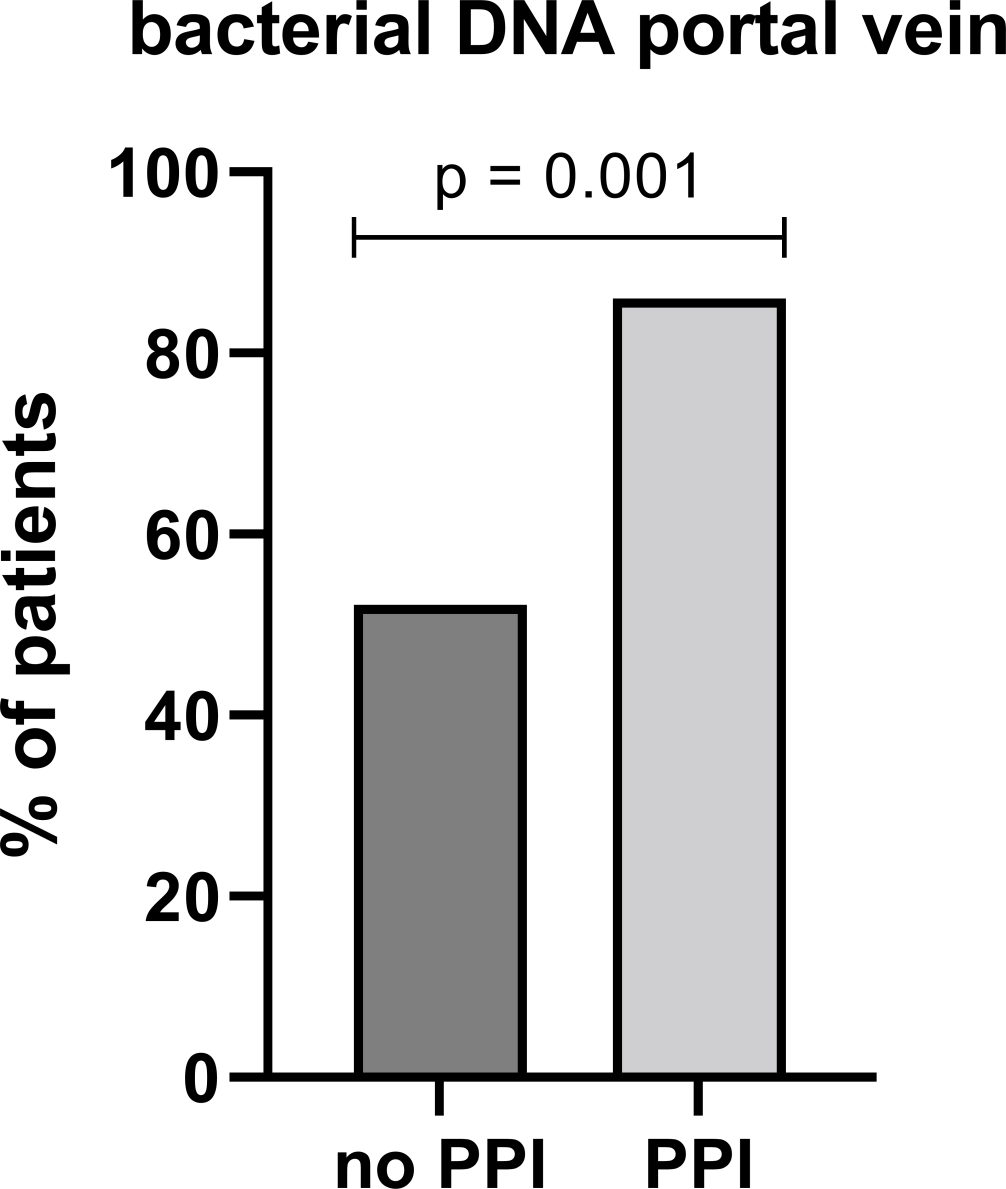
The rate of BT was higher in PPI-treated patients, as bacterial DNA was detectable significantly more frequently in the portal vein blood of patients in the PPI group compared to the no-PPI group (*n* = 49, 86.0 % vs. *n* = 12, 52.2 %, *p* = 0.001). Abbreviations: BT, bacterial translocation; DNA, deoxyribonucleic acid; PPI, proton pump inhibitor.

The 16S rRNA PCR revealed a high prevalence of BT in the included patients, as bacterial DNA was detectable in the portal vein blood of 61 patients (76.3%). Indeed, BT was strongly linked to PPI treatment, as bacterial DNA was detectable in 86.0% of patients in the PPI group (*n* = 49) compared to 52.2% of patients in the no-PPI group (*n* = 12, *P* = 0.001; Fig. 2). The corresponding individual gel electrophoresis results and repeated control amplification of negative samples are presented in Fig. S1 and S2. Importantly, the increase of BT in the PPI group was consistent when excluding patients receiving norfloxacin or rifaximin treatment (*n* = 41, 87.2% vs *n* = 7, 50.0%; *P* = 0.003) or patients with a history of spontaneous bacterial peritonitis (*n* = 41, 85.7% vs *n* = 8, 50.0%; *P* = 0.004).

### Microbiota composition in the portal vein blood

**Fig 3:**
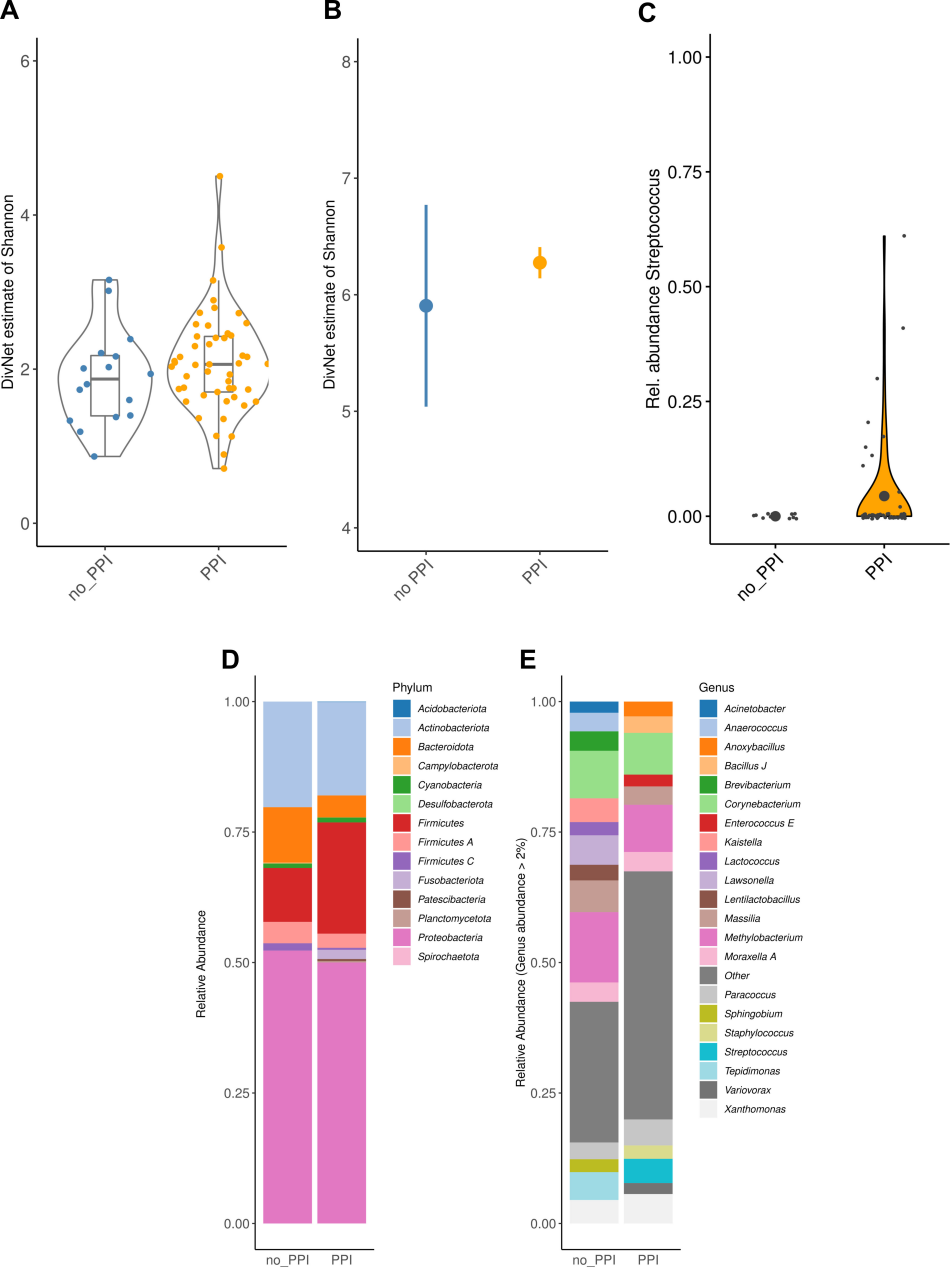
Microbiome analyses of the portal venous blood samples revealed significantly increased sample-wise and group-wise alpha-diversity (Shannon index) in the PPI group compared to the no-PPI group (A+B). Taxonomic analyses showed increased *Streptococcus* abundances in the PPI group (C). Otherwise, there were no significant differences on phylum or genus level between PPI group and no-PPI group (D+E).Abbreviations: PPI, proton pump inhibitor.

For microbiome analyses in the portal venous blood samples, samples with less than 1,000 contigs were removed from the analyses (*n* = 15), which left a total of 65 samples (*n* = 49, 75.4% with PPI treatment vs *n* = 16, 24.6% without PPI treatment). Alpha-diversity (Shannon index) was significantly increased in the PPI group versus the no-PPI group (sample-wise: 0.20, standard error 0.09, *P* = 0.036; group-wise 0.37, standard error 0.01; *P* < 0.001; Fig. 3A and B). There was no significant group difference in beta diversity, estimated using Aitchison distance (PERMANOVA: *R^2^=* 0.015, *P* = 0.671). Further, genus differences between the two patient groups were investigated based on previous observations of altered gut microbiota composition in PPI-treated individuals. Indeed, there was a significant increase of *Streptococcus* abundances in the PPI group (MaAsLin2: *P* = 0.066, likelihood ratio test: *P* = 0.040; Fig. 3C). Otherwise, there were no significant group differences on phylum or genus level (Fig. 3D and E).

### Parameters associated with BT in the study collective

To identify factors associated with BT other than PPI treatment, patient characteristics at the time of TIPS implantation were analyzed and stratified according to the detection of bacterial DNA in the portal vein. This showed that parameters of liver function were not clearly associated with BT in the present study collective (Table 2). Of note, BT was not significantly reduced in patients treated with norfloxacin or rifaximin, neither was it more frequent in patients with a history of spontaneous bacterial peritonitis.

**TABLE 2:** Patient characteristics stratified according to proof of bacterial DNA in the portal vein^*e*^

	No bacterial DNA (*n* = 19)	Bacterial DNA (*n* = 61)	*P*-value
**Age [years]**	61 (51– 72)	62 (57–69)	0.549
**Gender**			0.190
Female	3 (15.8)	19 (31.1)	
Male	16 (84.2)	42 (68.9)	
**Etiology**			0.476
Alcoholic	15 (78.9)	38 (62.3)	
HCV	1 (5.3)	7 (11.5)	
HBV	0	4 (6.6)	
NASH	1 (5.3)	8 (13.1)	
Other	2	4 (6.6)	
**Ascites**	17 (89.5)	55 (90.2)	0.930
**Prior HE**	3 (15.8)	14 (23.0)	0.505
**Prior SBP**	4 (21.1)	12 (19.7)	0.895
**TIPS indication**			0.127
Ascites^*a*^	15 (78.9)	54 (88.5)	
Secondary prophylaxis^*b*^	4 (21.1)	4 (6.6)	
Other^*c*^	0	3 (4.9)	
**PSG pre-TIPS [mmHg]**	19 (16–24)	18 (16–21)	0.679
**FIPS**	0.02 (−0.78–0.60)	0.18 (−0.27–0.65)	0.268
**FIPS risk group**			0.505
High-risk (≥ 0.92)	3 (15.8)	14 (23.0)	
Low-risk (< 0.92)	16 (84.2)	47 (77.0)	
**MELD**	13 (10–15)	13 (10– 16)	0.548
**Child-Pugh stadium**			0.538
A	4 (21.1)	7 (11.5)	
B	11 (57.9)	37 (60.7)	
C	4 (21.1)	17 (27.9)	
**Laboratory parameters**			
WCC [10^3^ /µL]	6 (4–9)	6 (5–8)	0.874
Platelets [10^3^ /µL]	134 (93–168)	114 (90–198)	0.808
Creatinine [mg/dL]	1.3 (0.9–1.9)	1.3 (0.9–1.9)	0.505
INR	1.2 (1.1–1.3)	1.2 (1.1–1.3)	0.982
Bilirubin [mg/dL]	1.0 (0.7–2.2)	1.1 (0.7–2.3)	0.861
Albumin [g/dL]	3.1 (2.7–3.8)	3.1 (2.8–3.4)	0.712
AST [U/L]	50 (30–66)	43 (30–64)	0.336
ALT [U/L]	24 (16–35)	26 (17–36)	0.890
Sodium [mmol/L]	136 (127–139)	135 (131–138)	0.870
**Medication**			
NSBB	9 (47.4)	20 (32.8)	0.248
Norfloxacin	3 (15.8)	7 (11.5)	0.620
Rifaximin	4 (21.1)	9 (14.8)	0.516
Lactulose	13 (68.4)	31 (50.8)	0.178
Antibiotic treatment^*d*^	5 (26.3)	17 (27.9)	0.895
PPI treatment	8 (42.1)	49 (80.3)	0.001

^*a*^ Refractory ascites including hepatic hydrothorax.

^*b*^ Secondary prophylaxis of variceal bleeding after failure of endoscopic and medicamentous treatment.

^*c*^ Preemptive TIPS within 72 hrs after variceal hemorrhage in two patients and portal decompression prior to planned abdominal surgery in one patient.

^*d*^ Systemic antibiotic treatment within four weeks prior to TIPS implantation.

^*e*^ ALT – alanine aminotransferase, AST aspartate aminotransferase, HBV/HCV – hepatitis B/C virus, DNA – deoxyribonucleic acid, FIPS – Freiburg Index of Post-TIPS Survival, GERD – gastroesophageal reflux disease, HE – hepatic encephalopathy, INR – international normalized ratio, MELD – Model for End-Stage Liver Disease, NASH – non-alcoholic steatohepatitis, NSBB – non-selective beta blocker, PPI – proton pump inhibitor, PSG – portosystemic pressure gradient, SBP – spontaneous bacterial peritonitis, TIPS – transjugular intrahepatic portosystemic shunt, WBC – white blood cells.

### Surrogate markers of BT in portal vein blood

Surrogate markers in the portal vein did not indicate BT in the included patients, as levels of LBP, sCD14, and TNF-alpha were not significantly different in patients with and without proof of bacterial DNA (Table 3). Interestingly, levels of LBP were lower in the PPI group compared to the no-PPI group, albeit the difference did not reach statistical significance. Otherwise, surrogate markers of BT were not relevantly different in PPI group versus no-PPI group.

**TABLE 3:** Surrogate markers of BT in the portal vein stratified according to proof of bacterial DNA and PPI treatment^*a*^

	Total of patients (*n* = 80)	No bacterial DNA (*n* = 19)	Bacterial DNA (*n* = 61)	*P*-value
**LBP** [ng/mL]	54.1 (45.9–68.9)	56.4 (47.3–68.7)	54.0 (45.5–69.3)	0.823
**sCD14** [ng/mL]	449 (294–1144)	447 (342–1175)	451 (291–1126)	0.615
**TNF-alpha** [pg/mL]	6.0 (3.6–11.0)	6.5 (2.3–11.2)	5.8 (3.7–10.3)	0.901
**IL-6** [pg/mL]	19.1 (10.9–37.9)	18.7 (10.9–40.1)	20.1 (11.1–37.3)	0.839
		**No-PPI (** ***n*** **= 23)**	**PPI (** ***n*** **= 57)**	
**LBP** [ng/mL]	54.1 (45.9–68.9)	59.6 (49.7–77.2)	52.7 (45.1–64.1)	0.051
**sCD14** [ng/mL]	449 (294–1144)	426 (301–1227)	466 (290–1123)	0.746
**TNF-alpha** [pg/mL]	6.0 (3.6–11.0)	5.8 (3.0–11.6)	6.2 (3.7–10.3)	0.832
**IL-6** [pg/ml]	19.1 (10.9–37.9)	20.9 (10.9–40.1)	17.8 (10.4–37.3)	0.655

^*a*^ IL-6 – interleukin-6, DNA – deoxyribonucleic acid, LBP – lipopolysaccharide binding protein, PPI – proton pump inhibitor, sCD14 – soluble cluster of differentiation 14, TNF-alpha – tumor necrosis factor alpha.

## DISCUSSION

The present study is the first to systematically investigate the impact of PPI treatment on BT in patients with cirrhosis. To allow direct detection of bacterial DNA translocated from the gut into the portal venous bloodstream, the study was performed in patients allocated to TIPS implantation, as this procedure involves catheterization of the portal vein. Of note, these patients represent a collective with advanced cirrhosis and recurrent complications of portal hypertension (30). Hence, it is not surprising that BT could be detected in a large proportion of the included patients, as the prevalence of BT increases with the progression of cirrhosis (31 – 33). Indeed, a comparison of patients with and without PPI treatment revealed that the prevalence of BT was significantly higher in PPI-treated patients. This finding is important since BT is associated with the development of complications and significantly impaired prognosis in patients with cirrhosis (20 – 22). Considering the fact that prescription of (long-term) PPI medication is a common practice in patients with cirrhosis, this result is also of high clinical relevance (1 – 3). In this context, it has to be noted that patients in the PPI group received norfloxacin treatment significantly more often. However, this is unlikely to introduce relevant bias in the analyses as (i) the effect of PPI treatment on BT was consistent when excluding patients with norfloxacin treatment and (ii) the prevalence of BT was not significantly lower in norfloxacin-treated patients in the present study. Of note, besides norfloxacin treatment, there were no significant differences between PPI and no-PPI with respect to the severity of chronic liver disease or medication that could serve to explain the higher prevalence of BT in the PPI group.

Microbiome analyses in the portal vein blood of the study collective revealed a significantly increased alpha-diversity in the PPI group. This indicates that PPI treatment is not only linked to a higher prevalence of BT *per se* but also to an increased load of bacterial species translocated from the gut into the portal vein. In this context, it is interesting to note that PPI treatment induces a decrease of alpha-diversity in the gut microbiota composition itself (13, 14). Taxonomic studies in the portal vein blood showed a significant increase of *Streptococcus* abundances on the genus level in the PPI group. It is worth noting that *Streptococcus* species, besides gram-negative bacteria, are a relevant pathogen in SBP and in bloodstream infections in patients with cirrhosis (34 – 36). Further, an abundance of *Streptococcus* is one of the central alterations in the gut microbiota composition that can be observed in PPI-treated individuals (13 – 16). This could be seen as an indicator for a role of PPI-induced intestinal dysbiosis in BT. However, as the gut microbiota composition was not analyzed in the present study, the interaction between PPI-induced changes of the gut microbiota and BT remains to be clarified in further studies.

In fact, parameters of liver function and stage of cirrhosis were not clearly associated with BT in the present study. Importantly, this result does not allow us to conclude that PPI treatment is sufficient for, let alone solely responsible for the development of BT. This is also highlighted by the significant prevalence of BT of more than 50.0% in the no-PPI group. BT results from an interplay of multiple factors related to chronic liver disease, such as portal hypertension, altered gut microbiota composition, and impaired immune barrier (19). As the included patients with and without PPI treatment were in a similar clinical stage of chronic liver disease (advanced cirrhosis with recurrent complications of portal hypertension), this relative homogeneity may serve to explain why only PPI therapy emerged as an effector of BT in our study collective.

In the present study, inflammatory markers previously proposed as surrogate markers of BT did not indicate the presence of bacterial DNA in the portal vein. This result is remarkable—interestingly, it is in conformity with another study that investigated BT in patients with TIPS implantation: Mortensen et al. also observed no correlation of indirect markers of BT with the detection of bacterial DNA in portal vein blood (37). As mentioned before, patients allocated to TIPS implantation represent the end-stage of cirrhotic portal hypertension. Thus, these findings may indicate that common surrogate markers are not suitable to detect BT in this subgroup of cirrhosis patients. However, this issue remains to be addressed specifically by further studies. Remarkably, levels of LBP were lower in patients with PPI treatment in comparison to patients without PPI treatment. An explanatory approach to this observation could be increased gram-positive BT in PPI-treated patients, as increased levels of LBP only reflect gram-negative BT (28).

The present study has some limitations that need to be discussed. The first is the limited number of 80 included patients. This sample size is comparable to previous studies investigating BT in patients with cirrhosis, and it allowed detection of significant group differences between patients with and without PPI treatment (20, 21, 37 – 40). However, in case of some potentially interesting sensitivity analyses, patient numbers were too small to allow sensible analyses. For example, this involves exploring the effect of PPI treatment among patients receiving norfloxacin or rifaximin treatment or a possible dose-dependency of PPI treatment and BT. Further, it cannot be fully excluded that the limited size of the study collective, especially in the no-PPI group, contributed to non-significance of differences in baseline characteristics between no-PPI group and PPI group. It also has to be mentioned that, while baseline parameters were similar in patients with and without PPI treatment, confounding by other parameters possibly affecting BT that were not accounted for, such as dietary habits, cannot be fully excluded. Another limitation of this study is the fact that bacterial DNA was assessed at a single time point and not studied longitudinally. Thus, episodes of BT could potentially have been missed. However, episodes of BT have been shown to last for 24–72 h, which increases the chance of detecting BT also by cross-sectional investigation (39). It could also be considered a limitation that no fecal microbiota analyses were performed. We acknowledge that this prevents to establish if increased BT in PPI-treated patients is a direct correlate of PPI-induced changes in the gut microbiota composition. Finally, as mentioned before, the included patients represent a collective of patients with advanced cirrhosis and recurrent complications of portal hypertension. Thus, it remains unclear if PPI treatment has similar negative effects in patients in earlier clinical stages of cirrhosis.

In conclusion, the present study reveals increased BT in patients with advanced cirrhosis and portal hypertension receiving PPI therapy. This finding could establish an important link between PPI treatment and adverse effects in cirrhosis. Follow-up studies are needed to further investigate the mechanisms by which PPI treatment promotes BT in patients with advanced cirrhosis. In any case, the present results argue for careful prescription of PPIs in patients with cirrhosis, as BT is associated with significantly impaired prognosis in these patients.

## SUPPLEMENTAL MATERIAL

File S1 mbio.00492-23-s0001.docx Statistical methods and data processing for microbiome analyses.

Figure S1 mbio.00492-23-s0002.tif Gel electrophoresis results.

Figure S2 mbio.00492-23-s0003.tif Repeated gel electrophoresis results for negative samples.

ASM does not own the copyrights to Supplemental Material that may be linked to, or accessed through, an article. The authors have granted ASM a non-exclusive, world-wide license to publish the Supplemental Material files. Please contact the corresponding author directly for reuse.

## References

[1] Dultz G , Piiper A , Zeuzem S , Kronenberger B , Waidmann O . 2015. Proton pump inhibitor treatment is associated with the severity of liver disease and increased mortality in patients with cirrhosis. Aliment Pharmacol Ther 41:459–466. doi:10.1111/apt.13061 25523381

[2] Cole HL , Pennycook S , Hayes PC . 2016. The impact of proton pump inhibitor therapy on patients with liver disease. Aliment Pharmacol Ther 44:1213–1223. doi:10.1111/apt.13827 27774677

[3] Sturm L , Bettinger D , Giesler M , Boettler T , Schmidt A , Buettner N , Thimme R , Schultheiss M . 2018. Treatment with proton pump inhibitors increases the risk for development of hepatic encephalopathy after implantation of transjugular intrahepatic portosystemic shunt (TIPS). United European Gastroenterol J 6:1380–1390. doi:10.1177/2050640618795928 PMC6206537 30386611

[4] Bajaj JS , Zadvornova Y , Heuman DM , Hafeezullah M , Hoffmann RG , Sanyal AJ , Saeian K . 2009. Association of proton pump inhibitor therapy with spontaneous bacterial peritonitis in cirrhotic patients with ascites. Am J Gastroenterol 104:1130–1134. doi:10.1038/ajg.2009.80 19337238

[5] Dam G , Vilstrup H , Watson H , Jepsen P . 2016. Proton pump inhibitors as a risk factor for hepatic encephalopathy and spontaneous bacterial peritonitis in patients with cirrhosis with ascites. Hepatology 64:1265–1272. doi:10.1002/hep.28737 27474889

[6] Nardelli S , Gioia S , Ridola L , Farcomeni A , Merli M , Riggio O . 2019. Proton pump inhibitors are associated with minimal and overt hepatic encephalopathy and increased mortality in patients with cirrhosis. Hepatology 70:640–649. doi:10.1002/hep.30304 30289992

[7] Tsai C-F , Chen M-H , Wang Y-P , Chu C-J , Huang Y-H , Lin H-C , Hou M-C , Lee F-Y , Su T-P , Lu C-L . 2017. Proton pump inhibitors increase risk for hepatic encephalopathy in patients with cirrhosis in a population study. Gastroenterology 152:134–141. doi:10.1053/j.gastro.2016.09.007 27639806

[8] Dam G , Vilstrup H , Andersen PK , Bossen L , Watson H , Jepsen P . 2019. Effect of proton pump inhibitors on the risk and prognosis of infections in patients with cirrhosis and ascites. Liver Int 39:514–521. doi:10.1111/liv.14012 30472808

[9] Merli M , Lucidi C , Di Gregorio V , Giannelli V , Giusto M , Ceccarelli G , Riggio O , Venditti M . 2015. The chronic use of beta-blockers and proton pump inhibitors may affect the rate of bacterial infections in cirrhosis. Liver Int 35:362–369. doi:10.1111/liv.12593 24836902

[10] Tergast TL , Wranke A , Laser H , Gerbel S , Manns MP , Cornberg M , Maasoumy B . 2018. Dose-dependent impact of proton pump inhibitors on the clinical course of spontaneous bacterial peritonitis. Liver Int 38:1602–1613. doi:10.1111/liv.13862 29675988

[11] Terg R , Casciato P , Garbe C , Cartier M , Stieben T , Mendizabal M , Niveyro C , Benavides J , Marino M , Colombato L , Berbara D , Silva M , Salgado P , Barreyro F , Fassio E , Gadano A , Study Group of Cirrhosis Complications of the Argentine Association for the Study of Liver Disease . 2015. Proton pump inhibitor therapy does not increase the incidence of spontaneous bacterial peritonitis in cirrhosis: a multicenter prospective study. J Hepatol 62:1056–1060. doi:10.1016/j.jhep.2014.11.036 25481567

[12] Sun S , Ye W , Zhao R , Hu J , Zhang X , Yang M , Zhao H , Sheng J . 2021. Proton pump inhibitor therapy does not affect prognosis of cirrhosis patients with acute decompensation and acute-on-chronic liver failure: a single-center prospective study. Front. Med 8:763370. doi:10.3389/fmed.2021.763370 PMC8631392 34859015

[13] Imhann F , Bonder MJ , Vich Vila A , Fu J , Mujagic Z , Vork L , Tigchelaar EF , Jankipersadsing SA , Cenit MC , Harmsen HJM , Dijkstra G , Franke L , Xavier RJ , Jonkers D , Wijmenga C , Weersma RK , Zhernakova A . 2016. Proton pump inhibitors affect the gut microbiome. Gut 65:740–748. doi:10.1136/gutjnl-2015-310376 26657899 PMC4853569

[14] Jackson MA , Goodrich JK , Maxan M-E , Freedberg DE , Abrams JA , Poole AC , Sutter JL , Welter D , Ley RE , Bell JT , Spector TD , Steves CJ . 2016. Proton pump inhibitors alter the composition of the gut microbiota. Gut 65:749–756. doi:10.1136/gutjnl-2015-310861 26719299 PMC4853574

[15] Horvath A , Rainer F , Bashir M , Leber B , Schmerboeck B , Klymiuk I , Groselj-Strele A , Durdevic M , Freedberg DE , Abrams JA , Fickert P , Stiegler P , Stadlbauer V . 2019. Biomarkers for oralization during long-term proton pump inhibitor therapy predict survival in cirrhosis. Sci Rep 9:12000. doi:10.1038/s41598-019-48352-5 31427714 PMC6700098

[16] Stadlbauer V , Komarova I , Klymiuk I , Durdevic M , Reisinger A , Blesl A , Rainer F , Horvath A . 2020. Disease severity and proton pump inhibitor use impact strongest on faecal microbiome composition in liver cirrhosis. Liver Int 40:866–877. doi:10.1111/liv.14382 31943691 PMC7187411

[17] Bajaj JS , Acharya C , Fagan A , White MB , Gavis E , Heuman DM , Hylemon PB , Fuchs M , Puri P , Schubert ML , Sanyal AJ , Sterling RK , Stravitz TR , Siddiqui MS , Luketic V , Lee H , Sikaroodi M , Gillevet PM . 2018. Proton pump inhibitor initiation and withdrawal affects gut microbiota and readmission risk in cirrhosis. Am J Gastroenterol 113:1177–1186. doi:10.1038/s41395-018-0085-9 29872220

[18] Lombardo L , Foti M , Ruggia O , Chiecchio A . 2010. Increased incidence of small intestinal bacterial overgrowth during proton pump inhibitor therapy. Clin Gastroenterol Hepatol 8:504–508. doi:10.1016/j.cgh.2009.12.022 20060064

[19] Wiest R , Lawson M , Geuking M . 2014. Pathological bacterial translocation in liver cirrhosis. J Hepatol 60:197–209. doi:10.1016/j.jhep.2013.07.044 23993913

[20] Zapater P , Francés R , González-Navajas JM , de la Hoz MA , Moreu R , Pascual S , Monfort D , Montoliu S , Vila C , Escudero A , Torras X , Cirera I , Llanos L , Guarner-Argente C , Palazón JM , Carnicer F , Bellot P , Guarner C , Planas R , Solá R , Serra MA , Muñoz C , Pérez-Mateo M , Such J . 2008. Serum and ascitic fluid bacterial DNA: a new independent prognostic factor in noninfected patients with cirrhosis. Hepatology 48:1924–1931. doi:10.1002/hep.22564 19003911

[21] El-Naggar MM , Khalil E-S-M , El-Daker MAM , Salama MF . 2008. Bacterial DNA and its consequences in patients with cirrhosis and culture-negative, non-neutrocytic ascites. J Med Microbiol 57:1533–1538. doi:10.1099/jmm.0.2008/001867-0 19018026

[22] Bruns T , Reuken PA , Stengel S , Gerber L , Appenrodt B , Schade JH , Lammert F , Zeuzem S , Stallmach A . 2016. The prognostic significance of bacterial DNA in patients with decompensated cirrhosis and suspected infection. Liver Int 36:1133–1142. doi:10.1111/liv.13095 26901072

[23] European Association for the Study of the Liver. Electronic address: easloffice@easloffice.eu, European Association for the Study of the Liver . 2018. EASL clinical practice guidelines for the management of patients with decompensated cirrhosis. J Hepatol 69:406–460. doi:10.1016/j.jhep.2018.03.024 29653741

[24] Rössle M . 2013. TIPS: 25 years later. J Hepatol 59:1081–1093. doi:10.1016/j.jhep.2013.06.014 23811307

[25] Thoendel M , Jeraldo PR , Greenwood-Quaintance KE , Yao JZ , Chia N , Hanssen AD , Abdel MP , Patel R . 2016. Comparison of microbial DNA enrichment tools for metagenomic whole genome sequencing. J Microbiol Methods 127:141–145. doi:10.1016/j.mimet.2016.05.022 27237775 PMC5752108

[26] Reimer-Taschenbrecker A , Künstner A , Hirose M , Hübner S , Gewert S , Ibrahim S , Busch H , Has C . 2022. Predominance of Staphylococcus correlates with wound burden and disease activity in dystrophic epidermolysis bullosa: a prospective case-control study. J Invest Dermatol 142:2117–2127. doi:10.1016/j.jid.2022.01.020 35149000

[27] Albillos A , de la Hera A , González M , Moya J-L , Calleja J-L , Monserrat J , Ruiz-del-Arbol L , Alvarez-Mon M . 2003. Increased lipopolysaccharide binding protein in cirrhotic patients with marked immune and hemodynamic derangement. Hepatology 37:208–217. doi:10.1053/jhep.2003.50038 12500206

[28] González-Navajas JM , Bellot P , Francés R , Zapater P , Muñoz C , García-Pagán JC , Pascual S , Pérez-Mateo M , Bosch J , Such J . 2008. Presence of bacterial-DNA in cirrhosis identifies a subgroup of patients with marked inflammatory response not related to endotoxin. J Hepatol 48:61–67. doi:10.1016/j.jhep.2007.08.012 17998145

[29] Aitchison J . 1982. The statistical analysis of compositional data. J Royal Statistical Society: Series B 44:139–160. doi:10.1111/j.2517-6161.1982.tb01195.x

[30] D’Amico G , Morabito A , D’Amico M , Pasta L , Malizia G , Rebora P , Valsecchi MG . 2018. Clinical states of cirrhosis and competing risks. J Hepatol 68:563–576. doi:10.1016/j.jhep.2017.10.020 29111320

[31] Pande C , Kumar A , Sarin SK . 2009. Small-Intestinal bacterial overgrowth in cirrhosis is related to the severity of liver disease. Aliment Pharmacol Ther 29:1273–1281. doi:10.1111/j.1365-2036.2009.03994.x 19302262

[32] Cirera I , Bauer TM , Navasa M , Vila J , Grande L , Taurá P , Fuster J , García-Valdecasas JC , Lacy A , Suárez MJ , Rimola A , Rodés J . 2001. Bacterial translocation of enteric organisms in patients with cirrhosis. J Hepatol 34:32–37. doi:10.1016/s0168-8278(00)00013-1 11211904

[33] Lin RS , Lee FY , Lee SD , Tsai YT , Lin HC , Lu RH , Hsu WC , Huang CC , Wang SS , Lo KJ . 1995. Endotoxemia in patients with chronic liver diseases: relationship to severity of liver diseases, presence of esophageal varices, and hyperdynamic circulation. J Hepatol 22:165–172. doi:10.1016/0168-8278(95)80424-2 7790704

[34] Llovet JM , Rodríguez-Iglesias P , Moitinho E , Planas R , Bataller R , Navasa M , Menacho M , Pardo A , Castells A , Cabré E , Arroyo V , Gassull MA , Rodés J . 1997. Spontaneous bacterial peritonitis in patients with cirrhosis undergoing selective intestinal decontamination. a retrospective study of 229 spontaneous bacterial peritonitis episodes. J Hepatol 26:88–95. doi:10.1016/s0168-8278(97)80014-1 9148028

[35] Choi S-H , Park H-G , Jun JB , Lee S-O , Choi S-H , Woo JH , Kim YS . 2009. Clinical characteristics and outcomes of pneumococcal bacteremia in adult patients with liver cirrhosis. Diagn Microbiol Infect Dis 63:160–164. doi:10.1016/j.diagmicrobio.2008.10.018 19150708

[36] Xie Y , Tu B , Xu Z , Zhang X , Bi J , Zhao M , Chen W , Shi L , Zhao P , Bao C , Qin E , Xu D . 2017. Bacterial distributions and prognosis of bloodstream infections in patients with liver cirrhosis. Sci Rep 7:11482. doi:10.1038/s41598-017-11587-1 28904387 PMC5597589

[37] Mortensen C , Karlsen S , Grønbæk H , Nielsen DT , Frevert S , Clemmesen JO , Møller S , Jensen JS , Bendtsen F . 2013. No difference in portal and hepatic venous bacterial DNA in patients with cirrhosis undergoing transjugular intrahepatic portosystemic shunt insertion. Liver Int 33:1309–1315. doi:10.1111/liv.12205 23763259

[38] Bellot P , García-Pagán JC , Francés R , Abraldes JG , Navasa M , Pérez-Mateo M , Such J , Bosch J . 2010. Bacterial DNA translocation is associated with systemic circulatory abnormalities and intrahepatic endothelial dysfunction in patients with cirrhosis. Hepatology 52:2044–2052. doi:10.1002/hep.23918 20979050

[39] Francés R , Benlloch S , Zapater P , González JM , Lozano B , Muñoz C , Pascual S , Casellas JA , Uceda F , Palazón JM , Carnicer F , Pérez-Mateo M , Such J . 2004. A sequential study of serum bacterial DNA in patients with advanced cirrhosis and ascites. Hepatology 39:484–491. doi:10.1002/hep.20055 14768002

[40] Such J , Francés R , Muñoz C , Zapater P , Casellas JA , Cifuentes A , Rodríguez-Valera F , Pascual S , Sola-Vera J , Carnicer F , Uceda F , Palazón JM , Pérez-Mateo M . 2002. Detection and identification of bacterial DNA in patients with cirrhosis and culture-negative, nonneutrocytic ascites. Hepatology 36:135–141. doi:10.1053/jhep.2002.33715 12085357

