# Supplementary material for: Proton pump inhibitor treatment aggravates bacterial translocation in patients with advanced cirrhosis and portal hypertension
Source: mBio. 2023 Aug 25;14(5):e00492-23. doi: 10.1128/mbio.00492-23 (PMC10653923; doi:10.1128/mbio.00492-23)
Supplement: File S1 — Statistical methods and data processing for microbiome analyses. [file mbio.00492-23-s0001.docx]

**Supplementary file 1: Data processing and statistical methods for microbiome analyses**

Amplicon sequencing, data processing and filtering

Sequencing reads (paired-end) were de-multiplexed and processed into amplicon sequencing variants (ASVs) using dada2^1^ (v1.20.0). Briefly, reads were truncated to 240bp (forward read) and 230bp (backward read), and the expected error (maxEE) was set to 1 for the forward and to 2 for the backward reads. Next, error rates were learned on 100 million bases for the forward and backward read data, and read pairs were merged. Merged sequences (contigs) were size selected (276 to 343bp), chimeric contigs were removed (92.91% of the contigs were non-chimeric), and the taxonomic assignment was performed by applying the IdTaxa^2^ algorithm (DECIPHER^3^ package v2.20.0) with GTDB^4^ r202 as a reference database. Afterward, ASVs without phylum assignment or assigned to the phylum *Cyanobacteria* were removed. Potential laboratory contaminations of the sequencing data were identified and discarded from the data using a frequency method (threshold 0.3, 20 ASVs identified as potential contaminants) and a prevalence method (threshold 0.3, 12 ASVs identified) as implemented in the decontam^5^ package (v1.16.0). In the final data set, only samples with at least 1,000 contigs were kept, leading to 65 samples with 674 ASVs. On average 5,396 contigs were present per sample (minimum 1,176, maximum 29,959).

Statistical analysis of amplicon sequencing data

Processed and filtered ASVs and covariate data were imported into R (v4.2.1) as a phyloseq^6^ object (v1.40.0). Alpha diversity (Shannon) was estimated sample and group-wise using DivNet^7^ (v0.3.7) and heterogeneity of total diversity was investigated by applying the *betta* function (breakaway^8^ v4.8.4) in the case of sample-wise estimates and the *testDiversity* function of the DivNet package in the case of group-wise estimates. Beta diversity was estimated using Aitchison distance^9^ (Euclidean distance of centered log-ratio transformed counts) and permutational multivariate analysis of variance using distance matrices (PERMANOVA) was used to analyze differences in beta diversity (*adonis2* function, vegan package v2.6-4, with 99,999 permutations).

Differentially abundant taxa were assessed using MaAsLin2^10^ (v1.10.0) with a linear modeling approach on log_10_-transformed total-sum scaled counts; results with p < 0.1 were considered statistically significant. To verify the finding on the genus *Streptococcus*, a likelihood ratio test approach (*H_0_: Streptococcus ~ 1*; *H_1_: Streptococcus ~ PPI_treatment*) as implemented in corncob^11^ (v0.3.0) was applied.

Supplementary References

1. McMurdie, P. J. *et al.* DADA2: High-resolution sample inference from Illumina amplicon data. *Nature Methods* 1–7 (2016) doi:10.1038/nmeth.3869.

2. Murali, A., Bhargava, A. & Wright, E. S. IDTAXA: a novel approach for accurate taxonomic classification of microbiome sequences. *Microbiome* **6**, (2018).

3. Wright, E., S. Using DECIPHER v2.0 to Analyze Big Biological Sequence Data in R. *The R Journal* **8**, 352 (2016).

4. Parks, D. H. *et al.* A standardized bacterial taxonomy based on genome phylogeny substantially revises the tree of life. *Nature Biotechnology* (2018) doi:10.1038/nbt.4229.

5. Davis, N. M., Proctor, D. M., Holmes, S. P., Relman, D. A. & Callahan, B. J. Simple statistical identification and removal of contaminant sequences in marker-gene and metagenomics data. *Microbiome* **6**, (2018).

6. McMurdie, P. J. & Holmes, S. phyloseq: An R Package for Reproducible Interactive Analysis and Graphics of Microbiome Census Data. *PLOS ONE* **8**, e61217 (2013).

7. Willis, A. D. & Martin, B. D. Estimating diversity in networked ecological communities. *Biostatistics* (2020) doi:10.1093/biostatistics/kxaa015.

8. Willis, A., Bunge, J. & Whitman, T. Improved detection of changes in species richness in high diversity microbial communities. *J. R. Stat. Soc. C* **66**, 963–977 (2017).

9. Aitchison, J. The Statistical Analysis of Compositional Data. *Journal of the Royal Statistical Society: Series B (Methodological)* **44**, 139–160 (1982).

10. Mallick, H. *et al.* Multivariable association discovery in population-scale meta-omics studies. *PLOS Computational Biology* **17**, e1009442 (2021).

11. Martin, B. D., Witten, D. & Willis, A. D. Modeling microbial abundances and dysbiosis with beta-binomial regression. *The Annals of Applied Statistics* **14**, 94–115 (2020).
