# Supplementary figures and images for: Proton pump inhibitor treatment aggravates bacterial translocation in patients with advanced cirrhosis and portal hypertension
Source: mBio. 2023 Aug 25;14(5):e00492-23. doi: 10.1128/mbio.00492-23 (PMC10653923; doi:10.1128/mbio.00492-23)

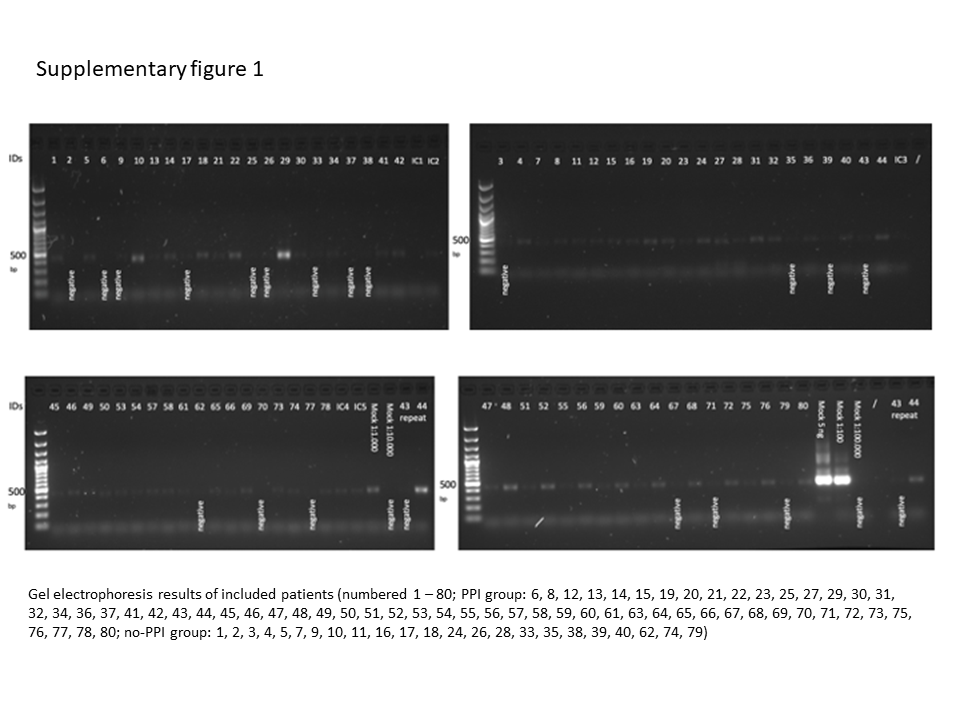

Supplement: Figure S1 — Gel electrophoresis results. [file mbio.00492-23-s0002.tif]

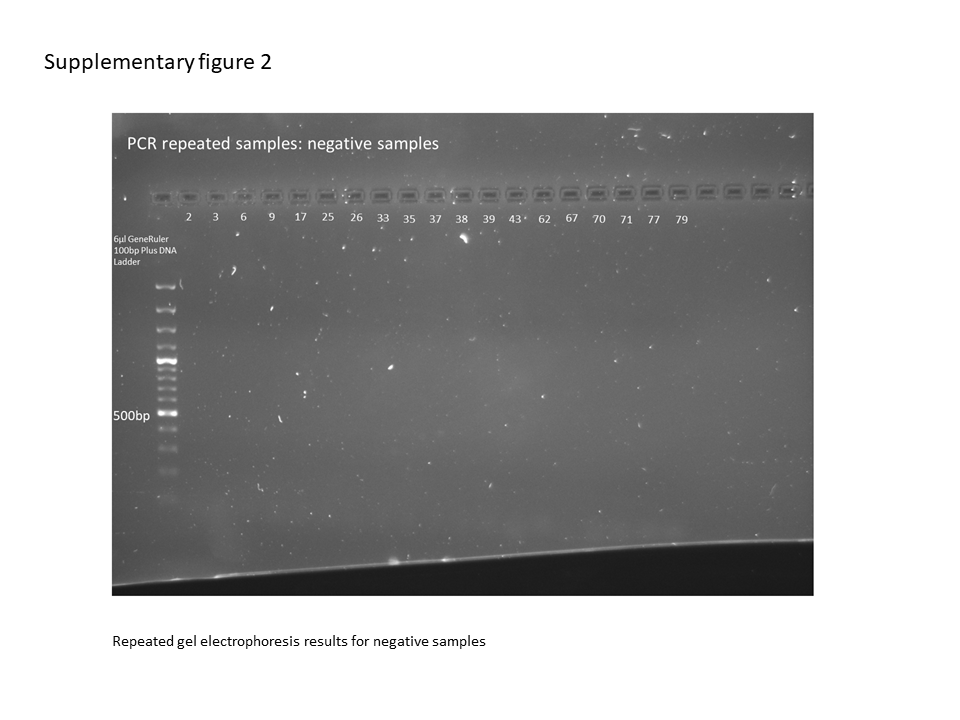

Supplement: Figure S2 — Repeated gel electrophoresis results for negative samples. [file mbio.00492-23-s0003.tif]
